# Investigation on the Essential Oils of the *Achillea* Species: From Chemical Analysis to the In Silico Uptake against SARS-CoV-2 Main Protease

**DOI:** 10.3390/life13020378

**Published:** 2023-01-30

**Authors:** Hossein Rabbi Angourani, Armin Zarei, Maryam Manafi Moghadam, Ali Ramazani, Andrea Mastinu

**Affiliations:** 1 Research Institute of Modern Biological Techniques (RIMBT), University of Zanjan, Zanjan 45371-38791, Iran; 2 Department of Chemistry, Faculty of Science, University of Zanjan, Zanjan 45371-38791, Iran; 3 Department of Molecular and Translational Medicine, University of Brescia, Viale Europa 11, 25123 Brescia, Italy

**Keywords:** *A. wilhelmsii* C. Koch., *A. tenuifolia* Lam., *A. millefolium* L., essential oil, gas chromatography, molecular docking, SARS-CoV-2

## Abstract

In this study, phytochemicals extracted from three different Achillea genera were identified and analyzed to be screened for their interactions with the SARS-CoV-2 main protease. In particular, the antiviral potential of these natural products against the SARS-CoV-2 main protease was investigated, as was their effectiveness against the SARS-CoV-1 main protease as a standard (due to its high similarity with SARS-CoV-2). These enzymes play key roles in the proliferation of viral strains in the human cytological domain. GC-MS analysis was used to identify the essential oils of the Achillea species. Chemi-informatics tools, such as AutoDock 4.2.6, SwissADME, ProTox-II, and LigPlot, were used to investigate the action of the pharmacoactive compounds against the main proteases of SARS-CoV-1 and SARS-CoV-2. Based on the binding energies of kessanyl acetate, chavibetol (m-eugenol), farnesol, and 7-epi-β-eudesmol were localized at the active site of the coronaviruses. Furthermore, these molecules, through hydrogen bonding with the amino acid residues of the active sites of viral proteins, were found to block the progression of SARS-CoV-2. Screening and computer analysis provided us with the opportunity to consider these molecules for further preclinical studies. Furthermore, considering their low toxicity, the data may pave the way for new in vitro and in vivo research on these natural inhibitors of the main SARS-CoV-2 protease.

## 1. Introduction

Iran is home to a significant share of plant species and endless natural habitats characterized by numerous special plants and centers of local endemic species [1]. *A. millefolium,* with the common name yarrow, is a member of the *Asteraceae* family and is a flowering plant [2] comprising 130 species, 19 of which are grow wild in Iran [1]. As far as the *Achillea* genus has existed across the world (from Asia and Europe to North America), it has been used in ancient life in traditional or folk medicine. Bumadaran is a common name for some species of *Achillea* in Persian culture [3].

The genus *Achillea* has a long history of use in traditional medicine as an anti-inflammatory, diaphoretic, anti-spasmodic, tonic, diuretic, and emmenagogic agent [3], and it has been used as a natural remedy (in Iranian traditional medicine) for the treatment of bleeding, headaches, respiratory infections, inflammation, spasmodic diseases, flatulence, dyspepsia [4,5], pneumonia, hemorrhaging, rheumatic pain, and wounds, and it is useful for liver disease and acts as a mild sedative [6]. Currently, the different medicinal functions of yarrow, such as its use as a spasmolytic, a choleretic, a wound-healing treatment, and an anti-inflammatory treatment, as well as its antifungal activities, have made it an important medicinal plant [3,7,8,9,10,11].

*A. willhelmsii* C. Koch (*Asteraceae*) possesses antioxidant, anti-inflammatory, antimicrobial, and antiulcerogenic properties, and it has been used to treat multiple diseases, as well as infections, hemorrhaging, pneumonia, rheumatic pain, and wounds [12]. The phytochemical characterization of the essential oil (EO) of *A. wilhelmsii* has been the subject of very few studies, most of which have focused on a small number of its molecules, including camphor, linalool, borneol, carvacrol, p-cymene, 1,8-cineole, and thymol, all of which show high potential for antioxidant activities [1].

*A. tenuifolia* Lam. (AT) is another member of the *Asteraceae* family that has been used in many cultures for more than 3000 years [13]. The attractive properties of AT extract include its anti-inflammatory, antitumor, antioxidant, and antimicrobial activities [14]. The antioxidant activity of AT is attributed to the presence of flavonoids and phenolic components [13,15]. Several studies have indicated that the biological function of AT is associated with its chemical composition. In addition, a correlation was found between the total phenol content and the antioxidant capacity of AT extract [16].

**Figure 1:**
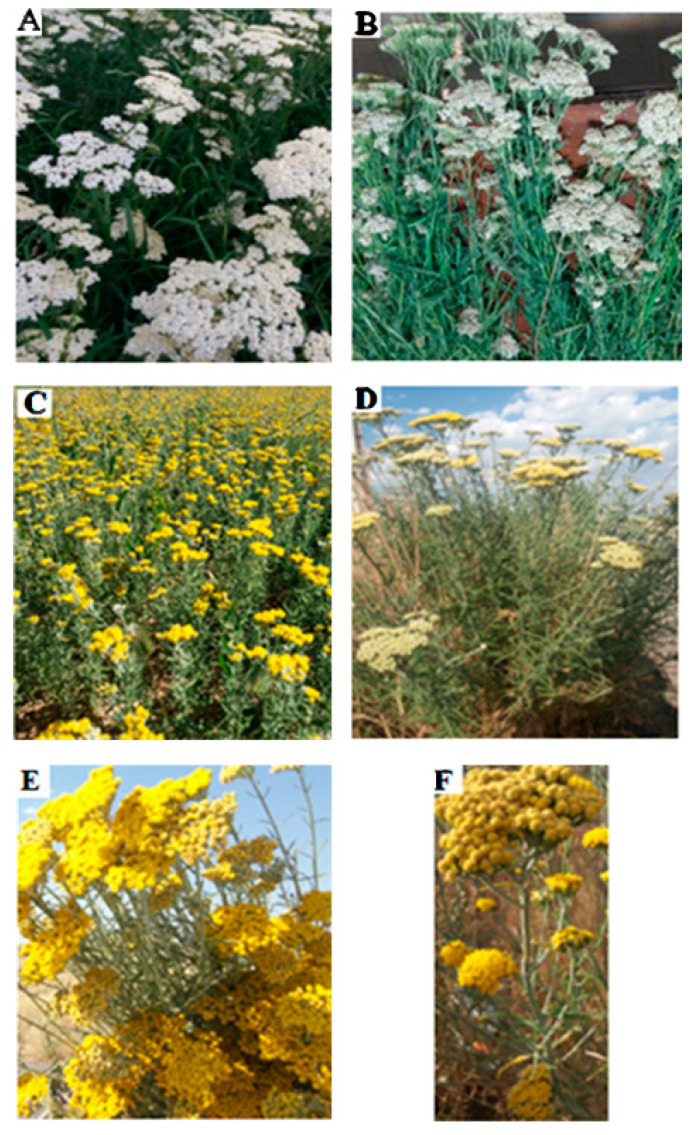
(**A**) Vegetative body of *A. millefolium* in its full flowering stage. (**B**) Vegetative body of a harvested plant drying. (**C**) Vegetative body of *A. wilhelmsii* in its full bloom stage. (**D**–**F**) Heads of branches and flowering limbs of the desert yarrow *A. tenuifolia* Lam.

The conservation of plant species (Figure 1) and innumerable natural habitats characterized by many unique plants and endemic centers is essential for pharmacological research [17,18,19,20,21,22,23,24,25,26]. Aromatic and medicinal plants produce a wide variety of volatile terpene hydrocarbons (aliphatic and cyclic) in addition to their corresponding oxygenated isoprenoid derivatives and analogs. Mixtures of these compounds, known as essential oils (EOs), can be isolated from diverse parts of plants by steam distillation and have excellent bioactivities, such as antimicrobial properties [2,27,28,29,30,31]. Moreover, EOs account for only a small portion of a plant’s composition; however, they determine the vital characteristics of aromatic plants [32]. As secondary metabolites, EOs involve complex mixtures of natural compounds with versatile organic structures that represent useful medicinal properties [33,34,35] which can be extracted from different parts of plant materials using classical and advanced techniques [36]. The composition of EOs is greatly influenced by diverse parameters, including the time and season of harvesting of the plants, the type of plant organs and the plant’s corresponding family, the plant’s geographical and climatic conditions, the plant’s physiological age, and the plant’s growth stage [10]. In addition, EOs and aromatic extracts are typically used as perfuming agents, pharmaceuticals, and food flavors, as well as in aromatherapy. Although the sophistication and complexity of EOs are undesirable properties for drug discovery, they are valuable in medical therapy and may be used as promising sources of novel drugs. Extracted compounds from EOs may be useful options against COVID-19, according to a recent study in which some signs of the inhibitory potential of EO constituents against the SARS-CoV-2 main protease have been highlighted [37].

Over the last few decades, viral diseases such as H1N1 flu, SARS-CoV-1, SARS-CoV-2, Ebola, and Mers have posed a serious threat to human society [38]. In 2019, a novel coronavirus disease (COVID-19) outbreak occurred in Wuhan, China. Since then, this viral disease has spread quickly worldwide, with millions of casualties [24,25,39]. This viral disease is caused by SARS-CoV-2 (syndrome acute respiratory CoronaVirus-2), which belongs to the beta-coronavirus family and shares a similarity of up to 79% to SARS-CoV-1, which underlies the next-generation sequencing technology. The SARS-CoV-2 3C-like protease (3CL^pro^), or the main protease (M^pro^), plays a vital role in the proteolytic processing of coronaviruses and creates other significant proteins for viral replication [40]. Hence, this protease may be the best target for therapeutic repositioning to identify promising antiviral drugs against SARS-CoV-2. Although pharmaceutical companies and global research groups have made efforts to find promising antiviral drugs to prevent the spread of COVID-19, some monoclonal antibodies, such as bamlanivimab–etesevimab [41] and a cocktail of casirivimab–imdevimab (REGEN-COV™) [42], and single monoclonal sotrovimab [43] have been authorized by the United States Food and Drug Administration (FDA) under emergency use authorization (EUA) for the treatment of patients (≥12 years of age) suffering from mild to moderate COVID-19 who are at high risk of progression to severe COVID-19 and/or hospitalization. However, this authorization was revoked on April 16, 2021 owing to the resistance of variants to bamlanivimab [44]. Remdesivir has been authorized for use in pediatric and adult patients (≥12 years of age) that require hospitalization, though it requires administration by injection or infusion in a therapeutic setting, with frequent monitoring [44,45]. In December 2021, two new oral antiviral agents, molnupiravir and nirmatrelvir/ritonavir (Paxlovid), gained emergency use authorization from the FDA for adult patients with mild to moderate COVID-19 who were at high risk of progression to severe COVID-19 under certain limitations [44,46,47]. Tocilizumab [48,49] and baricitinib [50,51] (both anti-inflammatory agents) have also gained EAU from the FDA for people of ≥ 18 years of age with COVID-19 who were admitted to a hospital [52].

The most promising drug target is the main protease of SARS-CoV-2 (M^pro^ or 3CL^pro^) because it plays a vital role in viral replication and transcription. Thus, the main axis of recent research has focused on the rapid development of SARS-CoV-2 M^pro^ inhibitors as drug candidates. Several techniques have been used for the discovery of SARS-CoV-2 M^pro^ inhibitors, including the high-throughput screening of structurally diverse compound libraries [53,54], drug repurposing [55,56], structure-based drug design [57,58], and in silico studies [59,60]. Despite considerable efforts, only one compound, nirmatrelvir/ritonavir (Paxlovid), has gained EUA by the FDA for adult patients with mild to moderate COVID-19 who are at a high risk of hospitalization [46], and a new compound (S-217622) is undergoing clinical trials (NCT05305547) [59].

Owing to their long history of folk use in Iranian traditional medicine and because of their low toxicity and excellent pharmacokinetics, the *Achillea* genera, alongside the reported biological activities of three Achillea species (*A. millefolium*, *A. wilhelmsii C*. Koch, and *A. tenuifolia* Lam), were the subject of this research. We wanted to address the question of whether the extracted compounds from three different EOs of the *Achillea* species can be the inhibitors, or have the potency to be a possible source of strong and/or effective inhibitors, of the SARS-CoV-2 3CL protease. The antiviral potential of the extracted compounds (from their EOs) against SARS-CoV-2 3CL^pro^ (and SARS-CoV-1 3CL^pro,^ as a standard) was investigated for the first time. Initially, the EOs of *A. millefolium*, *A. wilhelmsii* C. Koch, and *A. tenuifolia* Lam were obtained and their compounds were identified using GC-MS. The identified compounds were selected to investigate their antiviral potential against viral targets using AutoDcock 4.2.6. These compounds also showed low toxicity and very good pharmacokinetic properties in the SwissADME and ProTox-II evaluations. The compounds with the lowest binding energies (with the viral targets) were the best antiviral candidates against the viral protease and are worthy of further in vitro and in vivo analyses to clarify their antiviral potential.

## 2. Materials and Methods

### 2.1. Samples (Plant Materials)

The plants (three species of the Achillea genus: *A. millefolium*, *A. wilhelmsii* C. Koch, and *A. Tenuifolia* Lam) were collected during June and July 2021 from their natural habitat in the village of Homayoun in Zanjan province, Iran (elevation 1868 m, latitude east 48°28′35.36″, longitude 36°45′5.28″). A voucher specimen was maintained at the Research Institute of Modern Biological Techniques (RIMBT), University of Zanjan. Mr. Angourani deposited the botany herbarium voucher specimens under the following numbers: *A. millefolium,* number 1518; *A. tenuifolia* Lam, number 1522; and *A. wilhelmsii* C. Koch, number 1528. All samples were prepared from the whole flowers and leaves of the plants that were harvested at the full bloom stage of growth and then air-dried at room temperature in the shade.

### 2.2. Essential Oil Extraction

The essential oils from the flowers and leaves of the plants (*A. millefolium*, *A. wilhelmsii* C. Koch, and *A. tenuifolia* Lam) were extracted by hydrodistillation for approximately 4 h using a Clevenger apparatus in accordance with the British Pharmacopeia (1988) [61]. The finely cut plant material was fully submerged in distilled water (600 mL) in a round-bottom flask, which was then placed in a heating mantle. The condensed vapors were collected in a separating funnel and the resulting EO was separated, dehydrated with an appropriate amount of Na_2_SO_4_, filtered, and stored in brown vials at 4 °C.

### 2.3. GC-MS Analysis of the Essential Oil Components

The EOs were dehydrated with Na_2_SO_4_, and the samples were injected into the device under the thermal planning of the column. The compounds of the EOs were identified using the retention time (R.T) and the Kovats index (R.I) and by studying the mass spectra and checking these parameters with the standard compounds in the GC/MS library.

The EO extraction yields were estimated for 100 g of fresh plant material. The EOs were analyzed using gas chromatography-mass spectrometry (GC–MS). This device includes a gas chromatograph (model 7890B) and mass spectrometer (model 5977A) made by Agilent Company of America that was equipped with a split/splitless injection system and an ion bombardment ionization model (possessing the NIST and WILEY mass libraries). To analyze the fatty acids, an HP5-MS column with a length of 60 m, an inner diameter of 0.25 mm, and a thickness of 0.25 μm was used. The injection site, interface, and ionization site temperatures were set to 280, 290, and 250 °C, respectively. The temperature program of the column was triggered at an initial temperature of 60 °C, which remained constant for 5 min. The temperature reached 180 °C (with a slope of 15 °C/min) for 2 min before increasing to 280 °C (with a slope of 20 °C/ min), and it remained at this temperature for 10 min. The split ratio was set to 1 to 20 and the injection volume was half of a microliter.

### 2.4. Molecular Docking

The molecular and 3D structures of the targets (SARS-CoV-2 3CL^pro^: PDB code 6LU7 and SARS-CoV-1 3CL^pro^: PDB code 2H2Z) were extracted from the Protein Data Bank (www.rcsb.org (accessed on 1 January 2022)). To prepare the molecular structures of the natural compounds, their 2D chemical conformations were first drawn using the ChemSketch tool of the ACD/LAB package (www.acdlabs.com (accessed on 1 December 2022)) before transferring them to the Avogadro package to minimize energy and optimize their conformation via a steep algorithm.

#### Docking Studies

Molecular docking is a highly important sub-technique of molecular modelling that plays a significant role in computer-based drug design. The docking protocol was divided into two general stages using the AutoDock Autogrid package. Initially, blind docking was performed for all viral targets. Structures that bound to the active site of proteases with binding energies of >5.5 kcal/mol were chosen for the second stage (targeted docking). First, gastiger charges and polar hydrogen atoms were added to all ligands using the MGLtools package [62]. All bonds were set as active for ligands, and energetic maps were estimated for each atom type using Autogrid 4. For blind docking, the search space was set as large as all proteins were accessible for ligand binding. The active sites of the viral targets were set as search spaces for targeted docking. Finally, 250 runs of molecular docking according to the Lamarckian genetic algorithm were performed for ligands that met the conditions of the first step of the docking protocol [63]. Discovery studio 4.5 was used for the 3D visualizations of the results, and LigPlot^+^ V.2.2 was applied for the 2D presentations.

## 3. Results and Discussion

### 3.1. Achillea millefolium (Yarrow)

**Figure 2:**
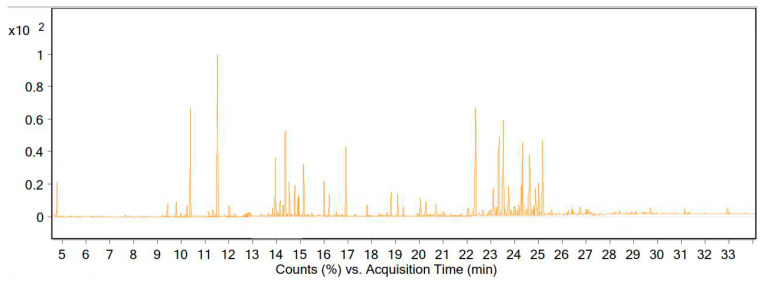
Chromatogram of the GC-MS analysis of the essential oil of *A. millefolium* from the Zanjan region.

The essential oils of the flowering and leafy branches of the dried vegetative body of *A. millefolium* were obtained via water distillation and a Clevenger apparatus, and the oil yield was 0.74% (content (% *v*/*w*)). The GC-MS results showed that the EO of the plant was composed of 105 compounds, of which 18 compounds accounted for 60.5% of the essential oil (Table 1 and Figure 2).

### 3.2. A. wilhelmsii C. Koch

**Figure 3:**
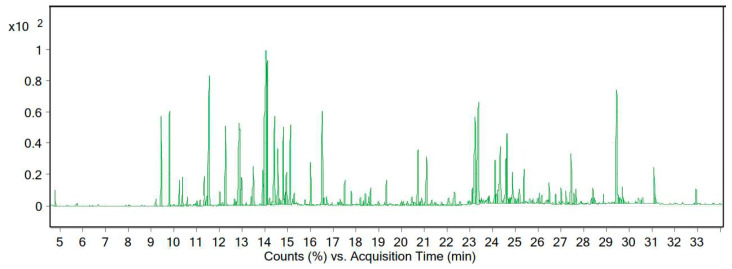
Chromatogram of the GC-MS analysis of the essential oil of *A. wilhelmsii* C.Koch from the Zanjan region.

The essential oil of *A. wilhelmsii* C. Koch was yellow in color, with a yield of 0.89% (% *v*/*w*). The GC-MS results revealed that the essential oil of this plant in the target area comprised 106 substances, of which 21 compounds represented 66.93% of the total essential oil (Table 2 and Figure 3).

### 3.3. A. tenuifolia Lam

**Figure 4:**
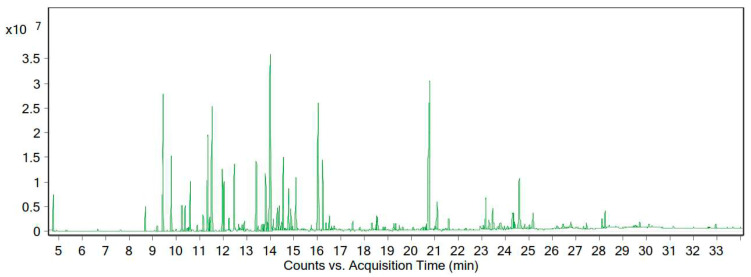
Chromatogram of the GC-MS analysis of the essential oil of *A. tenuifolia* Lam from the Zanjan region.

According to the GC-MS data, the essential oil of this plant contained 88 compounds. The essential oil of *A. tenuifolia Lam* was orange-yellow in color, with a yield of 0.82% (% *v*/*w*). The main chemical composition of the desert yarrow essential oil is presented in Table 3 and Figure 4. We identified 21 compounds that constituted 73.48% of the essential oil.

### 3.4. Docking Studies of SARS-CoV-2 3CL^pro^

Molecular docking is a potent approach used to elucidate protein and small-molecule interactions [24,25,64]. Among the identified compounds from the EOs of the three different types of yarrow tested against SARS-CoV-2 3CL^pro^, four natural inhibitors (Kessanyl acetate, Chavibetol (m-Eugenol), Farnesol, and 7-epi-β-Eudesmol) extracted from the EO of *A. millefolium* exhibited strong inhibitory effects against the viral target (Table 4). The binding energies of the proteases are listed in Table 4. It was clear that 3CL^pro^ (or the main protease) catalyzed the most important maturation cleavage phenomenon and also played a significant role in the viral replication of SARS-CoV-2, signifying its importance as a drug target [65].

**Figure 5:**
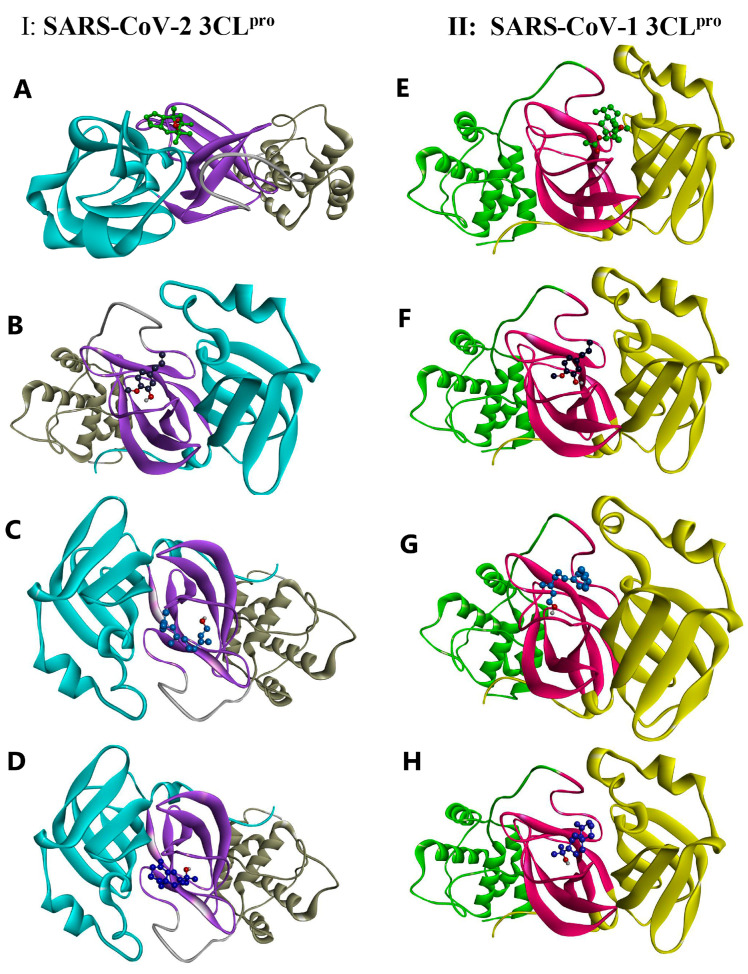
A 3D illustration of the interactions between the candidate compounds and the viral polymerases. (**A**–**D**) SARS-CoV-2 3CL^pro^: 6NUR. (**E**–**H**) SARS-CoV-1 3CL^pro^: 2H2Z. (**A**) Kessanyl acetate. (**B**) Chavibetol (m-Eugenol). (**C**) Farnesol. (**D**) 7-epi-β-Eudesmol.

As shown in Figure 5A (for the 2D version, see Figure S4), kessanyl acetate formed three H-bonds with the viral target: two with Ser 144 and one with the important residue Cys 145 (located in domain II and acting as a nucleophile in the initial step of the hydrolysis reaction in the catalytic region of the enzyme with the assistance of His 41 as a base catalyst) [65]; therefore, the interaction with this residue may have remarkably caused a malfunction in its enzymatic activities. Moreover, the hydrophobic interactions between kessanyl acetate and the important residues located in domains I and II [65], including His 41, Met 49, Leu 141, Asn 142, Gly 143, and Gln 189, can ratify the potent inhibitory effect of this natural product on the active site of the enzyme.

Cys 145 is located in the active site positioned in the oxyanion loop (residues 138–146), which, along with Gly 143, defines an oxyanion hole (via interactions between their amide groups and the carbonyl group of peptides), and, together with the β-strand segment (His163-Pro168), they are highly important for the preparation of active sites and substrate binding. Furthermore, residues near the loop segment play a significant role in expanding the active site [65]. Based on this information, it is worth pointing out that chavibetol (m-eugenol) binds to the active site of 3CL^pro^ through five strong hydrogen bonds with the residues Leu 141, Gly 143, Ser 144, and His 163, showing that this natural inhibitor may have high impairment and disruptive effects on the active site, and it may potentially disturb active site formation and accurate substrate binding (Figure 5B).

These disruptive effects on the active site formation and substrate binding of 3CLpro can be seen for the other two remaining natural inhibitors (Farnesol and 7-epi-β-Eudesmol) because each of them form H-bonds with Leu 141 and Ser 144, although the former shows an additional H-bond with His 163. Interestingly, both had hydrophobic interactions with Cys 145 and His 41, showing their strong antiviral effects on the catalytic function of this enzyme (Figure 5C,D). Taken together, these results show that the binding of the four natural inhibitors (extracted from *A. millefolium*) to the active site of 3CL^pro^ may disrupt substrate binding and active site formation, thereby impeding catalytic functions.

### 3.5. Docking Studies of SARS-CoV-1 3CL^pro^

To investigate the potency of the extracted natural products against other major proteases, we selected SARS-CoV-1 3CL^pro^ by using the same docking procedure. Based on the docking data, it could be asserted that four of the tested ligands showed higher binding energies (as they did against SARS-CoV-2 3CL^pro^). The 3CL proteases of SARS-CoV-1 and SARS-CoV-2 share approximately 96% sequence identity, indicating that they are similar to each other [66], and SARS-CoV-1 3CL^pro^ also possesses the three main domains, I, II, and III, and its catalytic dyad is composed of His 41 and Cys 145, in which its substrate-binding pocket is located in the cleft between I and II [67]. Interestingly, kessanyl acetate binds to this viral receptor through three H-bonds with Gly 143, Ser144, and Cys 145, which, in turn, may hinder the catalytic site and cause strong disorganization in the enzymatic function of the protease. In addition, hydrophobic interactions with other residues located in the active site (Leu 141 and Asn 142) can prove its high preventive effects on the catalytic site (Figure 5E).

As shown in Figure 5F, all interacting residues were located at the active site. Moreover, Chavibetol (m-eugenol) formed five strong hydrogen bonds with amino acid residues located in the catalytic region of domain II (Gly 143, Gly 143, Ser144, and His 163), and the active site entry may have been impaired [67], and the catalytic activities of such enzymes can be remarkably diminished. With regard to the interaction of SARS-CoV-2 3CL^pro^ with Farnesol and 7-epi-β-Eudesmol, it can be mentioned that both have formed H-bonds with Leu 141 and Ser 144, along with hydrophobic interactions with the significant catalytic dyad residues His 41 and Cys 145, indicating that these two natural inhibitors may possess highly potent activity for hindering the catalytic region of the protease, and they can potentially disrupt its enzymatic function (Figure 5G,H). Thus, these four natural inhibitors may have strong inhibitory effects on the active site of SARS-CoV-1 3CL^pro^ and could have a preventive and momentous disruptive impact on its catalytic function.

The four selected natural products were pharmacokinetically investigated using SwissADME. Interestingly, according to Table 5 and Table 6, all four natural inhibitors satisfied the drug-likeliness properties, indicating a high bioavailability score. Remarkably, none of them served as P-glycoprotein (P-gp) substrates, showing excellent oral availability of these ligands and indicating that they have no inhibitory or inductive effect on metabolizing enzymes [68,69]. More importantly, all showed high gastrointestinal absorption and could cross the blood–brain barrier, indicating that passing the cell membrane is vital for the components to enter the cells infected with SARS-CoV-2 and obtain successful access to the viral 3CL^pro^ of SARS-CoV-2. Consistent with our results, it should be noted that kessanyl acetate and farnesol possessed better pharmacokinetic characteristics, such as pharmacological and drug-likeliness properties. Although Kessanyl acetate did not interact with other medications, Farnesol and Chavibetol (m-eugenol) showed inhibitory effects on CYP1A2, which can be anticipated to interact with the metabolism of some typical anxiety or dispersive disorder drugs. Considering all of these properties, it can be postulated that these four naturally occurring compounds may be effectively absorbed, distributed, and diffused within the body. Finally, the toxicities of the four compounds were predicted using the ProTox-II online tool. As shown in Table 6, all compounds exhibited modest or light toxicity, and Farnesol and Kessanyl acetate showed higher LD50 values. Based on the results obtained in this study, it can be asserted that all four natural compounds (particularly kessanyl acetate and farnesol) may be the most desirable candidates for further in vitro and in vivo investigations of SARS-CoV-2.

## 4. Conclusions

The EO compounds from three different types of *Achillea* (*A. wilhelmsii* C. Koch, *A. tenuifolia* Lam, and *A. millefolium*) were explored using a gas chromatography device connected to a mass spectrometer. The antiviral potential of the identified compounds from the EOs of three different types of *Achillea* against SARS-CoV-2 3CL^pro^ (and SARS-CoV-1 3CL^pro^, as a standard) was computationally evaluated using Autodock 4.2.6. Our findings revealed that among the identified compounds (from the EOs of *Achillea* genera), four compounds extracted from the EO of *A. millefolium* (Kessanyl acetate, Chavibetol (m-Eugenol), farnesol, and 7-epi-β-Eudesmol) showed the highest binding energies and the maximum number in the clusters, revealing a high potential to demolish the active site of the proteases and ruin their catalytic functions by forming H-bonds with two important amino acid residues of CYS 145 and HIS 41. Furthermore, the ADME properties of the final compounds were determined to evaluate their pharmacokinetic characteristics. Our results revealed that kessanyl acetate and farnesol are the most suitable natural compounds with regard to drug pharmacokinetics.

Based on the findings of the present study, it should be mentioned that kessanyl acetate and farnesol are more potent inhibitors of 3CL-proteases than the other studied natural compounds, and they may be applied in future experimental studies to identify effective anti-SARS-CoV-2 compounds.

## Figures and Tables

**Table 1:** Major compounds in the essential oil of *A. millefolium* from the Zanjan region.

Number	Main Compounds	RT	RI ± (SD)	%
1	α-Pinene	9.428	929 ± (2)	2.21
2	Camphene	9.79	952 ± (2)	2.38
3	Sabinene	10.236	976 ± (3)	0.53
4	Eucalyptol (1,8-cineole)	11.54	1032 ± (2)	5.71
5	(+)-β-thujone	12.258	1103 ± (2)	2.55
6	Nonanal	12.856	1106 ± (3)	4.25
7	Trans-Verbenol	13.899	1143 ± (2)	0.88
8	Camphor	14.033	1147 ± (4)	9.87
9	(+)-2-bornanone	14.103	1152 ± (2)	5.59
10	Endo-borneol	14.421	1169 ± (2)	4.05
11	α-Terpineol	14.803	1189 ± (2)	4.46
12	(−)-Myrtenol	14.924	1208 ± (4)	0.87
13	Trans-p-Menth-1-en-3-ol	15.108	1213 ± (2)	2.58
14	Cis-Chrysanthenol acetate	15.999	1262 ± (3)	0.97
15	Rosefuran	16.501	1093 ± (2)	3.52
16	Caryophyllene	19.319	1418 ± (2)	0.64
17	D-Germacrene	20.706	1481 ± (2)	3.63
18	Spathulenol	23.218	1576 ± (2)	3.44
19	Caryophyllene oxide	23.377	1583 ± (2)	5.62
20	Isospathulenol	24.096	1638 ± (6)	1.3
21	7-Epi β-Eudesmol	24.611	1649 ± (2)	1.88
Total				66.93%

**Table 2:** Major compounds in the essential oil of *A. wilhelmsii* C. Koch from the Zanjan region.

Number	Main Compounds	RT	RI ± (SD)	%
1	α-Pinene	9.428	929 ± (2)	2.21
2	Camphene	9.79	952 ± (2)	2.38
3	Sabinene	10.236	976 ± (3)	0.53
4	Eucalyptol (1,8-cineole)	11.54	1032 ± (2)	5.71
5	(+)-β-thujone	12.258	1103 ± (2)	2.55
6	Nonanal	12.856	1106 ± (3)	4.25
7	Trans-Verbenol	13.899	1143 ± (2)	0.88
8	Camphor	14.033	1147 ± (4)	9.87
9	(+)-2-bornanone	14.103	1152 ± (2)	5.59
10	Endo-borneol	14.421	1169 ± (2)	4.05
11	α-Terpineol	14.803	1189 ± (2)	4.46
12	(−)-Myrtenol	14.924	1208 ± (4)	0.87
13	Trans-p-Menth-1-en-3-ol	15.108	1213 ± (2)	2.58
14	Cis-Chrysanthenol acetate	15.999	1262 ± (3)	0.97
15	Rosefuran	16.501	1093 ± (2)	3.52
16	Caryophyllene	19.319	1418 ± (2)	0.64
17	D-Germacrene	20.706	1481 ± (2)	3.63
18	Spathulenol	23.218	1576 ± (2)	3.44
19	Caryophyllene oxide	23.377	1583 ± (2)	5.62
20	Isospathulenol	24.096	1638 ± (6)	1.3
21	7-Epi β-Eudesmol	24.611	1649 ± (2)	1.88
Total				66.93%

**Table 3:** Major compounds in the essential oil of *A. tenuifolia* Lam from the Zanjan region.

Number	Main Compounds	RT	RI ± (SD)	%
1	α-Pinene	9.415	929 ± (4)	5.35
2	Camphene	9.765	951 ± (2)	2.45
3	β-Pinene	10.357	979 ± (3)	1.68
4	3-Carene	10.579	998 ± (3)	1.87
5	4-Carene	11.126	1011 ± (2)	0.53
6	o-Cymene	11.311	1025 ± (0)	3.93
7	D-Limonene	11.406	1031 ± (2)	0.57
8	Eucalyptol (1,8-Cineole)	11.502	1032 ± (2)	5.24
9	Gamma.-Terpinene	12.01	1060 ± (3)	1.7
10	Artemisia alcohol	12.449	1084 ± (3)	2.62
11	Trans-Borneol	13.974	1166 ± (2)	16.48
12	Endo-Borneol	14.364	1171 ± (3)	1.95
13	α-Terpineol	14.765	1182 ± (3)	1.62
14	Trans-p-Menth-1-en-3-ol	15.07	1195 ± (4)	2.15
15	Carvomenthenone	16.03	1254 ± (2)	6.78
16	Geranyl acetate	16.215	1379 ± (3)	2.18
17	Germacrene D	20.75	1481 ± (2)	10.49
18	Spathulenol	23.142	1576 ± (2)	1.34
19	Bornyl tiglate	23.454	1621 ± (3)	1.02
20	Epi-α-Cadinol	24.3	1640 ± (3)	0.85
21	α-Cadinol	24.573	1653 ± (4)	2.68
Total				73.48

**Table 4:** The results of the targeted molecular docking.

Chemical Name	Lowest Binding Energy (Kcal/mol) with 6NUR	Number of Run in Cluster	Lowest Binding Energy (Kcal/mol) with 2H2Z	Number of Run in Cluster
Kessanyl acetate	−6.23	87	−6.33	96
Chavibetol (m-Eugenol)	−5.69	123	−5.85	153
Farnesol	−5.78	115	−6.02	210
7-epi-β-Eudesmol	−5.47	73	−5.76	168

**Table 5:** Lipinski properties of the selected natural products.

Compound	Lipinski	Ghose	Veber	Egan	Muegge	Bioavailability Score
Kessanyl acetate	Yes	Yes	Yes	Yes	Yes	0.55
Chavibetol (m-Eugenol)	Yes	Yes	Yes	Yes	No	0.55
Farnesol	Yes	Yes	Yes	Yes	No	0.55
7-epi-β-Eudesmol	Yes	Yes	Yes	Yes	No	0.55

**Table 6:** Pharmacological properties and toxicity anticipation results for the four top natural products.

Compound	GI Absorption	BBB Permeation	P-glycoprotein Substrate	CYP1A2 Inhibitor	CYP2C19 Inhibitor	CYP2C9 Inhibitor	CYP2D6 Inhibitor	CYP3A4 Inhibitor	LD 50 mg/kg
Kessanyl acetate	High	Yes	No	No	No	No	No	No	2000
Chavibetol (m-Eugenol)	High	Yes	No	Yes	No	No	No	No	1230
Farnesol	High	Yes	No	Yes	No	No	No	No	5000
7-epi-β-Eudesmol	High	Yes	No	No	No	Yes	No	No	2000

## Data Availability

The availability of the data will be given upon appropriate request to the corresponding authors.

## Supplementary Materials

The following supporting information can be downloaded from: https://www.mdpi.com/article/10.3390/life13020378/s1, Figure S1, Chromatogram of the GC-MS analysis of *A. millefolium* essential oil from the Zanjan region; Figure S2, Chromatogram of the GC-MS analysis of the essential oil of *A. wilhelmsii* C. Koch from the Zanjan region; Figure S3, Chromatogram of the GC-MS analysis of essential oil of the vegetative body of *A. tenuifolia* Lam from the Zanjan region; Figure S4, A 2D illustration of the interactions between the candidate compounds and viral polymerases (I: SARS-CoV-2 3CL^pro^: 6NUR, and II: SARS-CoV-2 3CL^pro^: 2H2Z) ((a) kessanyl acetate, (b) chavibetol (m-eugenol), (c) Farnesol, and (d) 7-epi-β-Eudesmol).
