# Supplementary material for: Investigation on the Essential Oils of the Achillea Species: From Chemical Analysis to the In Silico Uptake against SARS-CoV-2 Main Protease
Source: Life (Basel). 2023 Jan 30;13(2):378. doi: 10.3390/life13020378 (PMC9967057; doi:10.3390/life13020378)
Supplement: Supplementary file 1 [file life-13-00378-s001.zip › life-2160000-supplementary.pdf]

Supplementary Meterials

Investigation on Essential Oils of *Achillea* Species: From Chemical Analysis to *In Silico* Uptake against SARS-CoV-2 Main Protease

Hossein Rabbi Angourani [1], Armin Zarei [2,*], Maryam Manafi Moghadam [2], Ali Ramazani [1,2,*], Andrea Mastinu [3,*]

[1]*Department of Biotechnology, Research Institute of Modern Biological Techniques (RIMBT), University of Zanjan, Zanjan 45371-38791, Iran*

[2]*Department of Chemistry, Faculty of Science, University of Zanjan, Zanjan 45371-38791, Iran*

[3]*Department of Molecular and Translational Medicine, University of Brescia, viale Europa 11, 25123 Brescia, Italy*

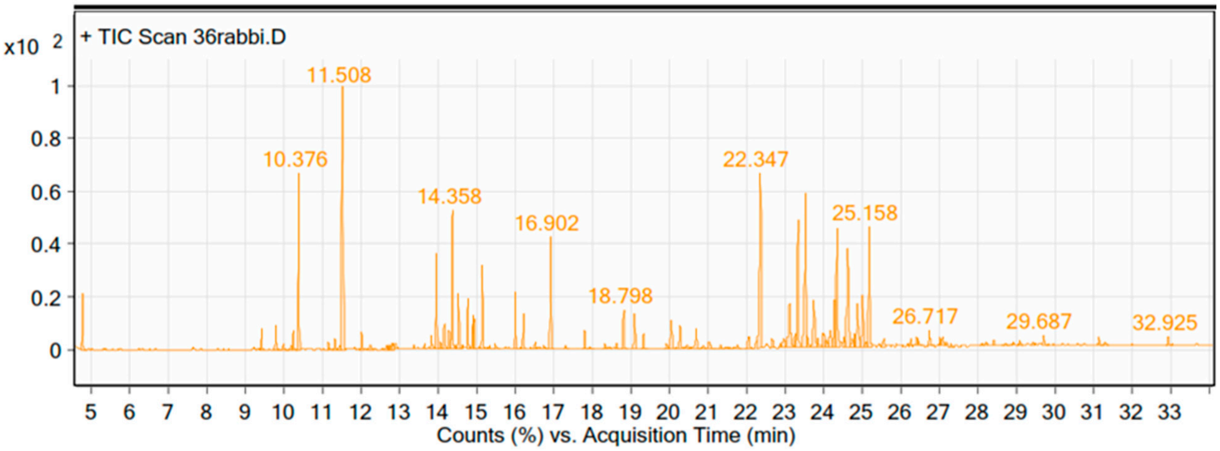

Figure S1. Chromatogram of GC-MS analysis of essential oil of *Achillea millefolium* in Zanjan region

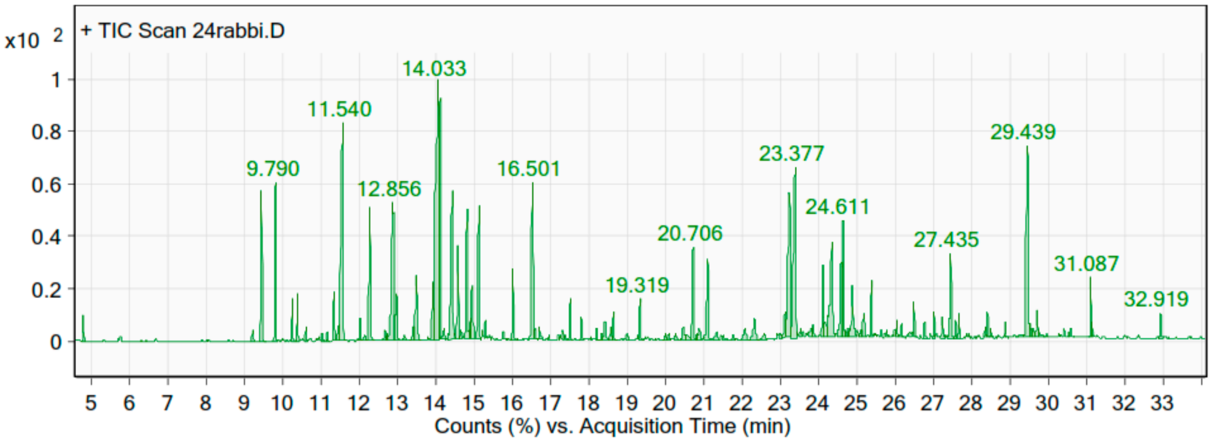

Figure S2. Chromatogram of GC-MS analysis of essential oil of *Achillea wilhelmsii* C. Koch in Zanjan region.

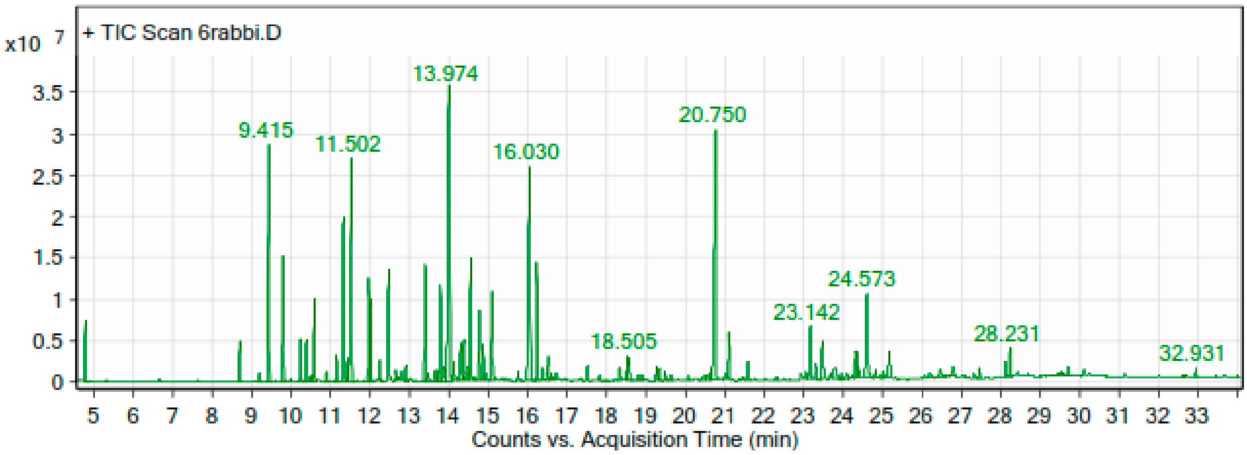

Figure S3. Chromatogram of GC-MS analysis of essential oil of vegetative body of *Achillea tenuifolia* Lam in Zanjan region

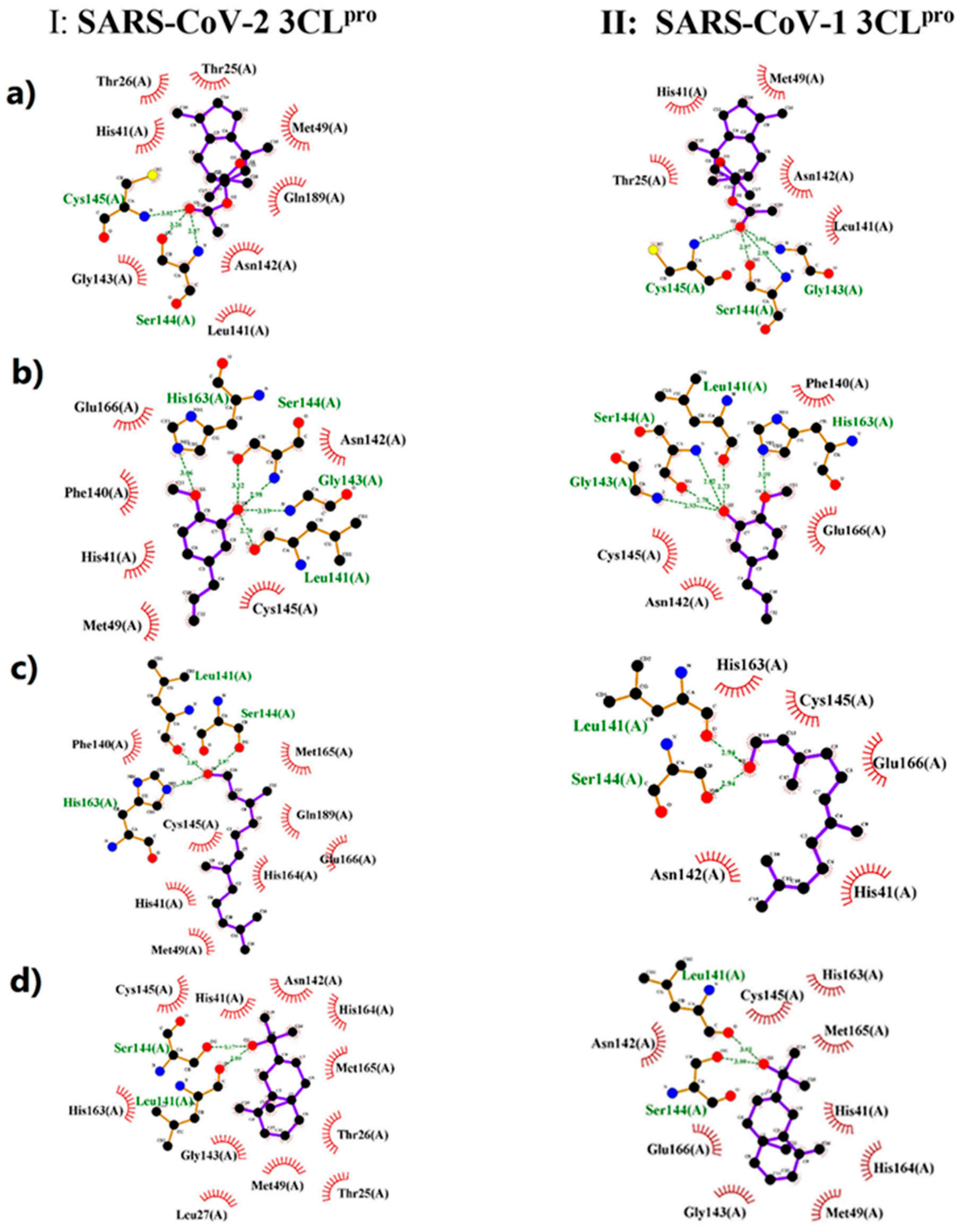

I: SARS-CoV-2 3CLpro
II: SARS-CoV-1 3CLpro
a)
Thr26(A)
Thr25(A)
His41(A)
Met49(A)
Cys145(A)
Gln189(A)
Gly143(A)
Asn142(A)
Ser144(A)
Leu141(A)
Met49(A)
His41(A)
Thr25(A)
Asn142(A)
Leu141(A)
Cys145(A)
Gly143(A)
Ser144(A)
b)
His163(A)
Ser144(A)
Glu166(A)
Asn142(A)
Phe140(A)
Gly143(A)
His41(A)
Leu141(A)
Met49(A)
Cys145(A)
Leu141(A)
Phe140(A)
Ser144(A)
His163(A)
Gly143(A)
Glu166(A)
Cys145(A)
Asn142(A)
c)
Leu141(A)
Ser144(A)
Phe140(A)
Met165(A)
His163(A)
Cys145(A)
Gln189(A)
Glu166(A)
His164(A)
His41(A)
Met49(A)
His163(A)
Cys145(A)
Leu141(A)
Glu166(A)
Ser144(A)
Asn142(A)
His41(A)
d)
Cys145(A)
His41(A)
Asn142(A)
His164(A)
Ser144(A)
Met165(A)
His163(A)
Leu141(A)
Thr26(A)
Gly143(A)
Met49(A)
Thr25(A)
Leu27(A)
Leu141(A)
Cys145(A)
His163(A)
Met165(A)
Asn142(A)
Ser144(A)
His41(A)
Glu166(A)
His164(A)
Gly143(A)
Met49(A)

Figure S4. 2D illustration of interactions between candidate compounds and viral polymerases; (I: SARS-CoV-2 3CL^{pro}: 6NUR, II: SARS-CoV-2 3CL^{pro}: 2H2Z): a) Kessanyl acetate, b) Chavibetol (m-Eugenol), c) Farnesol, d) 7-epi-β-Eudesmol.
